# Co-occurrence of the Cyanotoxins BMAA, DABA and Anatoxin-*a* in Nebraska Reservoirs, Fish, and Aquatic Plants

**DOI:** 10.3390/toxins6020488

**Published:** 2014-01-28

**Authors:** Maitham Ahmed Al-Sammak, Kyle D. Hoagland, David Cassada, Daniel D. Snow

**Affiliations:** 1 Environmental Health, Occupational Health, & Toxicology, Tropical Biological Researches Unit, College of Science, University of Baghdad, Baghdad 10071, Iraq; E-Mail: aitham@huskers.unl.edu; 2 School of Natural Resources, University of Nebraska, Lincoln, NE 68583, USA; E-Mail: khoagland1@unl.edu; 3 Nebraska Water Center and School of Natural Resources, University of Nebraska-Lincoln, Lincoln, NE 68583, USA; E-Mail: dcassada1@unl.edu

**Keywords:** BMAA, DABA, anatoxin-*a*, cyanobacteria, cyanotoxins, fish, plants, fresh water

## Abstract

Several groups of microorganisms are capable of producing toxins in aquatic environments. Cyanobacteria are prevalent blue green algae in freshwater systems, and many species produce cyanotoxins which include a variety of chemical irritants, hepatotoxins and neurotoxins. Production and occurrence of potent neurotoxic cyanotoxins β-*N*-methylamino-l-alanine (BMAA), 2,4-diaminobutyric acid dihydrochloride (DABA), and anatoxin-*a* are especially critical with environmental implications to public and animal health. Biomagnification, though not well understood in aquatic systems, is potentially relevant to both human and animal health effects. Because little is known regarding their presence in fresh water, we investigated the occurrence and potential for bioaccumulation of cyanotoxins in several Nebraska reservoirs. Collection and analysis of 387 environmental and biological samples (water, fish, and aquatic plant) provided a snapshot of their occurrence. A sensitive detection method was developed using solid phase extraction (SPE) in combination with high pressure liquid chromatography-fluorescence detection (HPLC/FD) with confirmation by liquid chromatography-tandem mass spectrometry (LC/MS/MS). HPLC/FD detection limits ranged from 5 to 7 µg/L and LC/MS/MS detection limits were <0.5 µg/L, while detection limits for biological samples were in the range of 0.8–3.2 ng/g depending on the matrix. Based on these methods, measurable levels of these neurotoxic compounds were detected in approximately 25% of the samples, with detections of BMAA in about 18.1%, DABA in 17.1%, and anatoxin-*a* in 11.9%.

## 1. Introduction

Cyanobacteria, or blue-green algae, are microscopic organisms occurring in both freshwater and marine environments. Cyanobacteria are a significant part of the phytoplankton and the periphyton in lacustrine environments and among the largest groups of microorganisms found worldwide [1]. Cyanobacteria can produce a wide variety of cyanotoxins including more than 80 known variations of microcystin heptapeptides and many lower molecular weight compounds [2]. Cyanobacteria blooms may form in aquatic environments, where large populations form thick blue-green accumulations at the water surface and shoreline. Multiple types of cyanotoxins have been found in or associated with 48% of cyanobacteria with most reports (95%) describing the occurrence of hepatotoxic microcystins.

Cyanobacteria are considered a growing problem in ecosystems worldwide because of toxins that they produce [3,4]. Cyanotoxins or phycotoxins are organic compounds produced by cyanobacteria in the environment with biological effects on other organisms [5,6]. Early reports of cyanotoxin poisoning in animals (cattle, horses, and dogs) occurred in 1878 [7]. Early reports of human poisoning from cyanotoxins in West Virginia, USA involved about 9000 people with gastroenteritis potential caused by drinking water contaminated with cyanotoxins [8]. Algal toxins include several groups of hepatotoxins, such as microcystins, neurotoxins such as anatoxin*-a*, and other compounds that can irritate the skin and gastrointestinal track such as lyngbyatoxin-*a* [4,9] (Table 1). Some cyanotoxins may accumulate in human and animals, causing a serious illness and poisoning [5].

**Table 1:** Some of the more important cyanotoxins and their effects on non-target organs.

Toxin	Non-target organ	Activity
Microcystins	Liver	Tumor Promoter
Nodularins	Liver	Carcinogenic
Cylinderospermopsin	Liver	Genotoxic
Anatoxin-*a*	Nervous System	Depolarizing neuromuscular blockers
Anatoxin-*a* (s)	Nervous System	Inhibits AchE
Saxitoxin	Nervous System	Na^+^ channel blocker
BMAA	Nervous System	Neurodegeneration
Lyngbyatoxin-*a*	Skin	Inflammatory agent
Aplysiatoxins	Skin	Inflammatory agent
Lipopolysaccharide	G.I.T.	Gastrointestinal irritant

**Figure 1:**
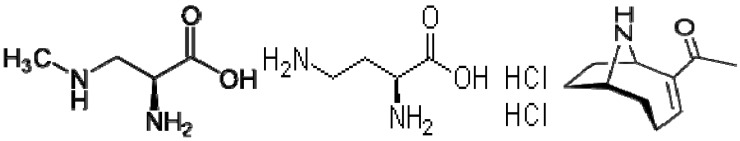
Structures of β-*N*-methylamino-l-alanine (BMAA), 2,4-diaminobutyric acid dihydrochloride (DABA) and anatoxin-*a*.

Several lower molecular weight cyanotoxins are of particular interest because they are studied less often, and have known or suspected neurotoxic effects. For example, β-*N*-methylamino-l-alanine (BMAA) (CAS number 15920-93-1) is a non-protein amino acid with the chemical name β-*N*-methylamino-l-alanine, molecular formula (C_4_H_10_N_2_O_2_) (Figure 1) and a relatively low molecular weight of 118.13 g/mol. It has been suggested that cellular exposure to BMAA may lead to neurologic damage in the brain and central nervous system of humans and animals, potentially contributing to one of several neurodegenerative diseases [10].

Polsky *et al*. [10] was the first to show BMAA neurotoxicity in extracts from cycad seeds used widely in Guam as a food and medicinal plant. Also, Karamyan and Speth [11] mentioned that BMAA can be incorporated into plant and animal proteins. BMAA has subsequently been detected in cyanobacterial blooms and laboratory isolates from marine and freshwater sources from localities worldwide including: Iraq, Qatar, Hawaii, China, United Kingdom, South Africa, Netherlands, and Sweden [12,13,14,15,16]. As a result, it has been hypothesized that BMAA can occur and bioaccumulate in other aquatic ecosystems where cyanobacteria are known to proliferate.

Studies have shown that BMAA occurs in zooplankton which can feed on cyanobacteria, and even higher BMAA levels were found in fish likely to feed on zooplankton [17,18]. Bioaccumulation of BMAA in aquatic organisms has been suggested in marine environments [19,20]. A particularly remarkable finding was the discovery of high BMAA levels in bottom-dwelling fish species (*S. maximus*, *T. quadricornis*, and *O. eperlanus*) and in water filter-feeding mollusks (*M. edulis* and *O. edulis*) [17]. BMAA was also detected in three aquatic animals’ species (zebra fish, brine shrimp, and the protozoan *Nassula sorex*) providing additional proof that BMAA can bioaccumulate in food chains, leading to potential human exposure [21]. BMAA was reported with a concentration as high as 7000 ng/g in South Florida samples that included water, cyanobacteria, fish (such as perch), and seafood (such as crab and shrimp) that are consumed by humans [22]. Detectable levels of BMAA was recently reported in shark fins, which have been used widely in the well-known “shark fin soup” in Florida [18].

Vranova *et al*. (2011) [23] showed that non-protein amino acids (NPAAs) play a significant role in plant, soil, and ecosystems functions. NPAAs are considered a store of organic nitrogen in many ecosystems, and are thought to be a toxins produced against both invertebrate and vertebrate animals, which could lead to serious human medical concerns. BMAA and DABA are NPAA toxins produced by cyanobacteria [24]. BMAA (β-*N*-methylamino-l-alanine) and DABA (2,4-diaminobutyric) are non-protein amino acids found in both marine and fresh waters [25]. Over the past 150 years, algal blooms, including those that are toxic, have caused problems worldwide, and now due to global warming these problems may accelerate [26].

Metcalf *et al*. [27] detected BMAA in British waterbodies along with other cyanotoxins such as microcystin, anatoxin-*a*, nodularin, and saxitoxin suggesting a health risk assessment of cyanobacterial BMAA in waterbodies. Cyanotoxins interfere with zebra fish (Danio rerio) embryo growth in an animal model system [28]. Salierno *et al*. [29] found that fish exposed to algal neurotoxins such as domoic acid, brevetoxin, and saxitoxin, suffered from severe loss of optic region activity in the brain. Microcystin are known to accumulate in liver, muscle, and viscera of fish, affecting their growth and survival rates [30].

DABA (Figure 1) is also a non-protein amino acid and BMAA-isomer. DABA is chemically known as 2,4-diaminobutyric acid, with the molecular formula C_4_H_10_N_2_O (Figure 1), CAS Number 1883-09-6, and molecular weight of 191.06 g/mol. DABA is thought to have the same toxicological character of BMAA but has not been extensively studied [31].

Anatoxin-*a* is an alkaloid compound with potent postsynaptic and depolarizing neuromuscular blockers [32]. The molecular formula is (C_10_H_15_NO) (Figure 1), CAS number 64285-06-9, and the molecular weight is 165.232 g/mol. Clinical signs of anatoxin-*a* exposure and neurotoxicity include muscular fasciculation, imbalance, and respiratory failure due to paralysis, leading to death [33,34,35]. Anatoxin-*a* was found to be produced by cyanobacteria isolated from Lake Biwa in Japan, along with homoanatoxin-*a* [36]. Anatoxin-*a* has been detected in Wisconsin and Florida waters and algal blooms with other cyanotoxins, such as cylindrospermopsins and microcystin-LR [33,37]. Anatoxin-*a* was also reported in cyanobacteria isolated from freshwater systems in Portugal, leading to suspicions of the growing health and ecological risk of its occurrence in fresh waters [38].

BMAA and DABA have different mechanisms of action in comparison to anatoxin-*a*. Anatoxin*-a* is a cyanotoxin with acute neurotoxicity. Anatoxin-*a* is produced by at least four genera of cyanobacteria and has been reported from North America, Europe, Africa, Asia, and New Zealand [39,40]. BMAA is produced by almost all cyanobacteria, such as the genera *Anabaena* and *Nostoc* which can live symbiotically in plant roots. The BMAA and DABA suggested mechanism of toxicity is though destruction of motor neurons in the brain, leading to neurodegenerative diseases likes Parkinson’s disease, Alzheimer’s, and ALS (Lou Gehrig’s disease) [13,20,41,42]. It was well explained and discussed how BMAA could be related to these neurodegenerative diseases by Papapetropoulos [42,43]. In one well-documented study, members of the Chamorro people on Guam who consumed the cycad seeds were found to suffer from ALS or Parkinsonism-dementia complex (PDC), which ultimately killed approximately 10% of Guam’s indigenous Chamorro population [42]. The traditional cuisine of the Chamorro people has also included flying fox bats, which feed on cycad flowers and fruits. Thus, it has been suggested that, “the plant and animal proteins provide unrecognized reservoirs for the slow release of this toxin” BMAA were detected to be bioaccumulated in cycads seeds and flying fox bats as produced by cyanobacteria that live endosymbiosis in the roots of tree cycads in Guam [20,24,44,45,46,47,48].

BMAA was also recently reported to occur in the brain tissue of nine Canadian’s Alzheimer patients [49]. Alternative ecological pathways likely occurs leading to bioaccumulation of BMAA in aquatic and terrestrial ecosystems [41,47,50]. Murch *et al*. [47] explained the mechanism of the slow release of biomagnified blue-green algal neurotoxins and neurodegenerative disease in Guam by suggesting that the BMAA neurotoxin produced by *Nostoc* can be concentrated in cycads seeds. Grinding the seeds for flour, the BMAA was concentrated over time in brain tissue causing death to Chamorro people who suffered from ALS. Cox *et al*. [41] showed that the algal neurotoxin BMAA can be produced by all known groups of cyanobacteria in both symbiotic and free-living forms. Their data showed that *Nostoc* can exist as symbionts with host plants such as cycads, some flowering plants such as *Gunnera*, and other cyanobacteria such as *Synechocystis* and *Anabaena*, found in U.S. fresh waters.

### Nebraska Lakes and Impoundments

Cyanotoxins have also been show to co-occur with drinking water taste-and-odor compounds, such as geosmin and 2-MIB in 91% of blooms sampled in a regional study of Midwestern lakes and impoundments [51]. In Nebraska, algal toxin problems have become a more growing health concern with reported cases of pet, livestock and wildlife deaths, cases of GIT illness, and cutaneous rashes from contaminated lakes and ponds [52]. In all these cases, acute exposure to toxins of cyanobacteria is suspected to be the primary cause.

Nebraska lakes and reservoirs appear to be particularly prone to cyanobacteria blooms. Beginning in 2004, cyanotoxins were regularly monitored by the Nebraska Department of Environmental Quality. Several lakes included in this monitoring program are annually reported to be a health alert issue in Nebraska [53]. Pawnee Reservoir #34, Fremont Lake #20, Carter Lake, Swan Creek #5A, Willow Creek Reservoir #46, and others have exceeded 20 µg/L of microcystin. WHO health advisory concentration of microcystins as determined by immunoassay [54]. A total of about 700 water samples were collected for monitoring algal toxin levels from 34 public lakes and reservoirs between 2004 and 2010. The number of lakes monitored has increased since then [53,54]. According to the Nebraska Water Monitoring program report for 2010, lakes/reservoirs within Nebraska are under weekly monitoring from May 1 to September 30 each year. Weekly monitoring usually includes samples of fish and water for bacteria, and for algal toxins, namely microcystins [54]. Health alerts are triggered in public lakes and reservoirs by NDEQ when the microcystin level reaches 20 µg/L, as measured by immunoassay. In 2004, health alerts were issued for 17 lakes/reservoirs (50% of lake samples), 12 lakes in 2005 (35%), six lakes in 2006 (12%), and six lakes in 2007 (13%) [53,54].

The present study was conducted to evaluate the potential for occurrence of neurotoxic cyanotoxins in Nebraska lakes and reservoirs, as well as associated plant and fish tissue collected during two summers, when cyanobacteria blooms are prevalent. The study employed a new detection method using derivatization combined with chromatography and fluorescence or mass spectrometry to simultaneously detect groups of cyanotoxins in biological and environmental samples [55]. Few wide-spread monitoring studies of neurotoxic cyanotoxins have been conducted because of the difficulties in measuring low molecular weight and water soluble organics at trace levels in complex environmental matrices. The present study provides a snap shot of the occurrence of these compounds in lake water and in aquatic organisms likely to serve as a route of human exposure.

## 2. Results and Discussion

Out of 387 environmental and biological samples including water, fish, and aquatic plants, 94 samples (24.3%) contained detectable levels of cyanotoxins measured using either HPLC/FD or LC/MS/MS (Table 2.).

**Table 2:** Summary of environmental samples collected from 2009 to 2010.

Sample	2009	2010	Total
water	31	36	67
Fish	115	133	248
Aquatic plants	36	36	72
Total samples collected between 2009 and 2010 =			387

### 2.1. Water Samples

**Figure 2:**
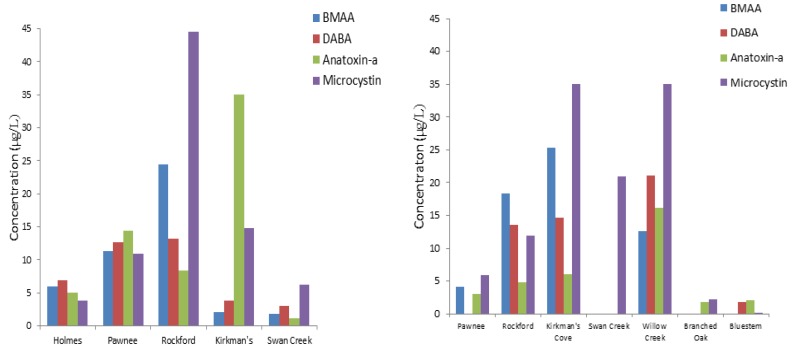
Comparison of BMAA, DABA, anatoxin-*a* concentrations measured in this study with total microcystin levels reported for reservoirs sampled in 2009 (**left**) and 2010 (**right**). Microcystin concentrations are measured by immunoassay and taken from the Nebraska Department of Environmental Quality (NDEQ) website.

Variable concentrations of cyanotoxins were measured in water samples collected from eight reservoirs between 2009 and 2010, and were not detected in control reservoirs. Conestoga reservoir was reported by NDEQ to have a level of 7.8 µg/L of microcystin in 2009 (Figure 2, Table 3). In 2009, the BMAA concentrations ranged from below detection up to 24.5 µg/L in Rockford reservoir, Gage County. DABA concentrations ranged up to 13.2 µg/L in Rockford, Gage County, while anatoxin-*a* concentrations ranged up to 35.0 µg/L in Kirkman’s Cove, Richardson County (Table 3). In 2010, BMAA concentrations ranged up to 25.3 µg/L in Kirkman’s Cove, Richardson County. Measured DABA concentrations ranged up to 21.1 µg/L in Willow Creek, Pierce County. BMAA and DABA were detected in 13 water samples in 2009 and in 12 water samples in 2010. BMAA and DABA were found together in all water samples as DABA is BMAA isomer as reported previously [25]. Anatoxin-*a* was detected in 13 water samples in 2009 and in 18 water samples in 2010 at concentrations ranging up to 35 µg/L. Anatoxin-*a* concentrations ranged from below detection up to 16.1 µg/L in Willow Creek, Pierce County (Table 3). Though microcystins have been regularly monitored over the past seven years, BMAA, DABA, and anatoxin-*a* have not been previously reported in Nebraska reservoir waters. This is the first report of BMAA, DABA, and anatoxin-*a* not just in Nebraska, but in any Midwestern aquatic ecosystem [55]. Jonasson *et al*. [17] hypothesized that BMAA can bioaccumulate in ecosystems, and may transfer within major food webs. NDEQ regularly monitors lake/reservoir water quality during the summer months, and makes health reports when microcystins exceed 20 µg/L (ppb) because it is now known that multiple cyanobacteria species have the ability to produce BMAA and DABA [41]; there may be a relationship between these compounds and microcystin levels (Figure 2).

Conestoga (Table 3) was reported to have detectable levels of microcystins by the ELISA test but did not have detectable levels of the other cyanotoxins measured in this study. The species of algae that produce microcystin are not necessarily the same that produce BMAA, DABA, or anatoxin-*a*. The NDEQ report on biweekly monitoring of microcystins, and issue a health alert preventing the public to get involved in any recreation activity when microcystin exceeds 20 µg/L (ppb). NDEQ monitors weekly or twice-monthly selected public lakes that are popular recreational sites usually during the summer season starting from May through September of each year. The occurrence of anatoxin-*a* has been previously reported in surface water [33,37]. Hedman *et al*. [37] report that anatoxin-*a* was associated with the production of microcystin by cyanobacteria in Wisconsin water. Anatoxin-*a* was found in four samples out of 74 analyzed samples with a concentration range between 0.068 and 17.5 µg/L. Microcystin was detected in 36 of 74 samples with a concentration ranging from 0.12 to 7.6 µg/L. These anatoxin-*a* levels are comparable to levels measured in this study. Williams *et al*. [33] report on the occurrence of cyanobacterial toxins in the Florida freshwater system with anatoxin-*a* concentrations of 0.05–7.0 µg/L.

**Table 3:** Cyanobacteria neurotoxin concentrations (µg/L) measured in water samples from Nebraska reservoirs from 2009 and 2010. HPLC/FL detection limits were 5.0 µg/L for BMAA, 4.3 for BOAA, 7.0 for DABA, 6.0 for anatoxin-*a*, and 5.0 µg/L for microcystin ^†^. ND = Not Detected; ^†^ Microcystin concentrations determined by Abraxis ELISA method are from the Nebraska Department of Environmental Quality website.

Reservoir	County	Dates sampled (number of samples)	BMAA (µg/L) 2009/2010	DABA (µg/L) 2009/2010	Anatoxin-*a* (µg/L) 2009/2010	Microcystins ^†^ (µg/L) 2009/2010
Holmes	Lancaster	9.7.09(3)/8.6.10(3)	6.0/ND	6.9/ND	5.0/ND	3.8/0.98
Pawnee	Lancaster	10.28.09(2)/8.20.10(3)	11.3/4.2	12.7/ND	14.4/3.04	11.0/5.9
Wagon Train	Lancaster	10.6.09(3)/10.26.10(3)	ND/ND	ND/ND	ND/ND	ND/ND
Stage Coach	Lancaster	10.7.09(3)/10.26.10(3)	ND/ND	ND/ND	ND/ND	ND/ND
East Twin	Seward	10.9.09(3)/10.25.10(3)	ND/ND	ND/ND	ND/ND	ND/ND
Rockford	Gage	8.3.09(3)/8.22.10(3)	24.5/18.3	13.2/13.6	8.4/4.8	44.5/11.9
Kirkman’s Cove	Richardson	8.3.09(3)/8.8.10(3)	2.1/25.3	3.9/14.7	35.0/6.0	14.8/35.0
Swan Creek	Saline	9.10.09(2)/9.9.10(3)	1.8/ND	3.0/ND	1.2/ND	6.3/21.0
Conestoga	Lancaster	10.8.09(02)/8.23.10(3)	ND/ND	ND/ND	ND/ND	7.8/0.13
Willow Creek	Pierce	8.30.09(3)/10.1.10(3)	ND/12.6	ND/21.1	ND/16.1	15.1/35.0
Branched Oak	Lancaster	9.20.010(2)/9.20.10(3)	ND/ND	ND/ND	ND/1.84	0.92/2.2
Bluestem	Lancaster	9.25.09(2)/9.8.10(3)	ND/ND	ND/1.78	ND/2.08	18.3/0.15

Osswald *et al*. [38] did not detect free anatoxin-*a* in samples from nine surface water reservoirs in Portugal, though 13 of 22 isolated cyanobacteria strains from these same environments were found to produce anatoxin-*a* in the laboratory. Seasonal variation of anatoxin-*a* levels has been reported in other studies and may coincide with algal activity [17].

### 2.2. Fish Tissue

A variety of fish representative of Midwestern reservoirs were collected to investigate the effect of trophic level on neurotoxin occurrence. Fish samples were prepared and analyzed for both free and bound cyanotoxins. Detectable levels of BMAA, DABA, or anatoxin-*a* were found primarily in bottom feeding fish or the fish that depend on aquatic plants and algae as their main food source, such as catfish, drum, and carp. Previous investigators have also noted that these compounds are more likely to accumulate in bottom-dwelling fish [17,56,57]. In 2009, the highest bound BMAA levels were detected in carp from Kirkman’s Cove with a concentration of 1.39 µg/g, while the lowest concentration were in bluegill from Willow Creek at a concentration of 0.056 µg/g. Free BMAA concentrations ranged between the highest level of 0.416 µg/g in bass from Pawnee and the lowest level was 0.103 µg/g in carp from Kirkman’s Cove. The highest bound DABA recorded was 1.16 µg/g in white crappie from Rockford, and the lowest level was 0.167 µg/g in Walleye from Pawnee. The highest free DABA detected was 0.364 µg/g in bass fish from Pawnee, while the lowest was 0.239 µg/g in carp from Kirkman’s Cove.

In 2010, the highest concentration of bound BMAA detected in carp collected from Rockford with a concentration of 2.57 µg/g, and the lowest was 0.476 µg/g in carp from Willow Creek. The highest free BMAA concentration detected in white Crappie from Kirkman’s Cove was 0.327 µg/g, while the lowest was 0.06 µg/g in a White crappie from Rockford. The highest bound DABA recorded was 1.53 µg/g in carp from Rockford Lake, and the lowest was 0.129 µg/g in a walleye from Rockford. The highest free DABA recorded was 0.213 µg/g in a walleye fish from Rockford, while the lowest was 0.0216 µg/g in a carp from Willow Creek (Table 4).

**Table 4:** HPLC/FD results showing cyanotoxin concentrations (µg/g) measured in fish samples collected in reservoirs in Nebraska, 2009 and 2010.

Reservoir	Species (# indiv. collected)	Year	BMAA (µg/g) bound free		DABA (µg/g) bound free		Anatoxin- *a* (µg/g) bound free	
Kirkman’s Cove	Carp (1)	2009	1.39	0.103	0.31	0.239	ND	ND
	Carp (1)	2010	0.8	0.102	0.184	0.158	ND	ND
	White crappie (2)	2010	0.6	0.327	ND	ND	ND	ND
Pawnee	Bass (3)	2009	0.3	0.416	0.2	0.364	ND	ND
	Shad (3)	2009	0.099	0.196	0.254	ND	ND	ND
	Walleye (2)	2009	0.604	0.25	0.167	ND	ND	ND
	White crappie (2)	2009	0.16	0.332	0.18	ND	ND	ND
Rockford	Bass (3)	2009	0.31	0.27	1.04	ND	ND	ND
	Catfish (2)	2009	0.32	0.254	0.28	ND	ND	ND
	White crappie (2)	2009	ND	ND	1.16	ND	ND	ND
	Carp (1)	2010	2.57	0.057	1.53	0.167	ND	ND
	Walleye (1)	2010	0.67	0.095	0.129	0.213	ND	ND
	White crappie (1)	2010	0.629	0.06	0.229	0.0218	ND	ND
Swan Creek	Carp (1)	2009	0.58	ND	0.2	ND	ND	ND
	Wiper (1)	2009	ND	0.227	ND	ND	ND	ND
Willow Creek	Bass (1)	2009	0.72	0.117	ND	ND	ND	ND
	Bluegill (3)	2009	0.056	0.36	ND	ND	ND	ND
	Carp (3)	2010	0.476	0.101	0.159	0.0216	ND	ND

In general, BMAA were detected in 31 fish tissue samples, while DABA were detected in 26 fish samples. In all fish samples that were collected between 2009 and 2010, no anatoxin-*a* was detected (Table 4), because anatoxin-*a* may not have the same accumulation pathway as BMAA and DABA throughout the tissues of fish. BMAA was found in fish and invertebrates collected from the Baltic Sea during the summer season of 2007–2008. The level of BMAA detected was between 0.008 and 0.059 µg/g, measured using LC-MS/MS [17]. These results show that BMAA occurred in fish tissue and at levels similar to those measured in this study. Turbot, herring, and common whitefish were found to have measureable levels of BMAA, and are also used for human consumption. Jonasson *et al*. [17] found the highest BMAA concentrations in bottom-dwelling fishes comparable to this study. BMAA has been reported in South Florida invertebrates including pink shrimp, blue crab, scrawled cowfish, with concentrations ranging between 34 and 6976 µg/g in tissue samples collected during the spring and summer of 2007. Most recently, Mondo *et al*. [18] found BMAA in fins of all seven different species of shark collected from South Florida. They used both HPLC/FD and LC/MS/MS and their results showed concentrations of BMAA between 144 and 1836 ng/g. Shark fins are popularly used in shark fin soup in Florida. These previous studies indicate a wide range of BMAA in animal tissue, similar to the present study of Nebraska reservoirs [24,48,50].

Ibelings *et al*. [58] indicated that anatoxin-*a* is less likely to occur in fish and seafood, and even less in samples taken from freshwater, which is consistent with a lack of detections for anatoxin-*a* in any of the fish samples collected in this study.

### 2.3. Aquatic Plants

In general, both in 2009 and 2010, BMAA, DABA, and anatoxin-*a* were detected in 15 aquatic plant samples; these results were the first to be reported for all three cyanotoxins in Nebraska freshwater ecosystems. In our cyanobacterial neurotoxin investigation in reservoirs, the principle genus of aquatic plant was *Myriophyllum* (water milfoil). Cyanobacteria are known to live in endosymbiotic relationships with other higher plants such as *Gunnera*, and water milfoil [41]. On the other hand, cyanotoxins can occur in aquatic plant tissue through direct exposure and absorption from surrounding contaminated environment, as shown in several studies [19,59,60].

Cyanotoxins were measured in 30 of 72 aquatic plant samples collected during 2009–2010. Free and bound cyano-neurotoxin was determined in aquatic plants. For 2009, the highest level of bound BMAA detected in aquatic plants was 6.7 µg/g taken from Rockford, and the lowest detected level was 4.5 µg/g from Holmes. The highest free BMAA detected in aquatic plant was 3.51 µg/g from Pawnee, and the lowest was 1.86 µg/g from Rockford. The highest bound DABA recorded in aquatic plant was 3.37 µg/g from Kirkman’s Cove and the lowest was 1.61 µg/g from Holmes. Only one lake sample showed a detectable level of free DABA—1.96 µg/g from Swan Creek. The highest bound anatoxin-*a* detected was in Swan Creek, with a concentration of 8.01 µg/g, and the lowest was 1.47 µg/g in Rockford. The highest level of free anatoxin-*a* was 0.61 µg/g in Holmes, and the lowest was 0.26 µg/g in Rockford (Table 5).

**Table 5:** Cyano-neurotoxin concentrations (µg/g) measured using HPLC/FL in aquatic plant samples from 12 reservoirs in Nebraska in 2009 and 2010.

Reservoir	BMAA (µg/g) 2009		BMAA (µg/g) 2010		DABA (µg/g) 2009		DABA (µg/g) 2010		Anatoxin-*a* (µg/g) 2009		Anatoxin-*a* (µg/g) 2010	
	Free	Bound	Free	Bound	Free	Bound	Free	Bound	Free	Bound	Free	Bound
Bluestem	ND	ND	ND	ND	ND	ND	ND	ND	ND	ND	ND	ND
Branched Oak	ND	ND	ND	ND	ND	ND	ND	ND	ND	ND	ND	ND
Conestoga	ND	ND	ND	ND	ND	ND	ND	ND	ND	ND	ND	ND
East Twin	ND	ND	ND	ND	ND	ND	ND	ND	ND	ND	ND	ND
Holmes	ND	ND	ND	4.5	ND	ND	ND	1.61	ND	ND	0.61	4.44
Kirkman’s Cove	6.68	12.7	ND	4.68	ND	2.59	ND	3.37	0.24	8.05	ND	4.61
Pawnee	ND	0.48	3.51	6.56	ND	1.9	ND	2.11	ND	0.18	0.36	2.47
Rockford	13.4	7.53	1.86	6.7	ND	2.06	ND	2.16	0.22	2.36	0.26	1.47
Stagecoach	ND	ND	ND	ND	ND	ND	ND	ND	ND	ND	ND	ND
Swan Creek	ND	3.61	2.61	6.37	ND	7.29	1.96	3.26	0.36	2.71	0.38	8.01
Wagon Train	ND	ND	ND	ND	ND	ND	ND	ND	ND	ND	ND	ND
Willow Creek	4.53	7.07	ND	ND	2.19	8.31	ND	ND	0.42	3.25	ND	ND

In 2010, the highest bound BMAA detected was 12.7 µg/g from aquatic plants collected from Kirkman’s Cove, and the lowest was 0.48 µg/g in Pawnee. While the highest free BMAA detected was 13.4 µg/g in Rockford, and the lowest was 4.53 µg/g from Willow Creek. The highest bound DABA detected in aquatic plant was 8.31 µg/g from Willow Creek, and the lowest detected level was 1.9 µg/g in Pawnee. Meanwhile, the highest free DABA were founded just in one lake sample (Willow Creek) with a concentration of 2.19 µg/g. The highest bound anatoxin-*a* concentration in aquatic plants was 8.05 µg/g from Kirkman’s Cove, and the lowest was 0.18 µg/g from Pawnee. Meanwhile, the highest free anatoxin-*a* level was 0.42 µg/g from Willow Creek, and the lowest was 0.22 µg/g from Rockford (Table 5).

BMAA was first isolated from cycad seeds (*Cycas circinalis*) and considered a cycad toxin [61], but Cox *et al*. [24] discovered that BMAA was actually produced by cyanobacteria that live symbiotically in roots of cycad trees. BMAA was found in the free amino acids of animal and plant tissues [24,48,50]. Duncan *et al*. [62] found BMAA in the female gametophyte tissue or the endosperm of cycads seeds, ranging from 0.29 to 1 mg/g of dry weight. BMAA and DABA were also found to be concentrated within the reproductive system of tree cycads (*Cycas micronesica*) [20]. Murch *et al*. [50] explained the mechanism for slow release of cyanobacterial neurotoxins. In their study, they found BMAA released from the cyanobacterial genus *Nostoc* found in the cycad root BMAA was also found in leaf tissue (738 µg/g), outer seed layer (48 µg/g), the seed sarcotesta (89 µg/g), and in the female gametophyte (81 µg/g), all in bound form. BMAA can be produced by almost all cyanobacteria in freshwater and marine ecosystems [41,59]. BMAA was found in cyanobacteria-plant symbioses in freshwater plants like water fern (*Azolla filiculoides*) (2 µg/g), and in *Gunnera kauaiensis* (4 µg/g) [24]. *Gunnera* and *Azolla* were found in Nebraska [63].

Metcalf *et al*. [27] detected BMAA in all 12 cyanobacteria blooms, scums, and mats collected from 1990 to 2007, with a concentration ranging between 8 and 287 µg/g, with anatoxin-*a* and other cyanotoxins (microcystin, nodularin, and saxitoxin) in 10 of the 12 samples taken from British water-bodies. This present study also suggests that BMAA and anatoxin-*a* can be associated with other cyanotoxins in water, which is consistent with these results. BMAA, DABA, and anatoxin-*a* in aquatic plant samples generally co-occur in lakes with detectable levels of these toxins. Cyanobacteria provide energy and nitrogen to the higher plant [64], and then via an unknown pathway, these cyanotoxins may also accumulate in plant tissues.

**Table 6:** Comparison of concentrations measured using LC/MS/MS and HPLC/FD for selected water, fish, and aquatic plant samples (water concentrations in µg/L, fish and aquatic plants (in µg/g)).

Reservoir	Sample Type		HPLC/FD			LC/MS/MS		
		Year	BMAA	DABA	Anatoxin-*a*	BMAA	DABA	Anatoxin-*a*
Holmes	Water	2009	6.0	6.9	5.0	15.6	18.4	19.7
Kirkman’s Cove	Water	2009	2.1	3.9	35	6.52	10.8	35.7
Pawnee	Water	2009	11.3	12.7	14.4	39.6	37.0	29.8
Conestoga	Water	2010	ND	ND	ND	ND	ND	ND
Willow Creek	Water	2010	12.6	21.14	16.1	23.6	32.3	30.1
Rockford	Water	2009	24.5	13.2	8.4	27.5	30.0	33.2
Swan Creek	Water	2009	1.8	3.0	1.2	10.9	18.9	5.2
Pawnee	Water	2010	4.2	ND	3.04	5.01	ND	13.5
Kirkman’s Cove	Water	2010	25.3	14.7	6.0	29.0	35.1	18.6
Rockford	Water	2010	18.3	13.6	4.8	24.0	33.1	22.6
Swan Creek	Plant/free	2010	2.61	1.96	0.38	2.38	1.28	0.711
Pawnee	Plant/free	2010	3.51	ND	0.36	3.41	ND	1.45
Pawnee	Plant/bound	2010	6.56	2.11	2.47	19.0	1.85	3.23
Holmes	Plant/bound	2010	4.5	1.61	4.44	2.69	1.09	7.92
Kirkman’s Cove	Plant/bound	2009	12.7	2.59	8.05	15.25	11.7	10.18
Conestoga	Plant/bound	2010	ND	ND	ND	ND	ND	ND
Wagon Train	Fish/bound	2009	ND	ND	ND	ND	ND	ND
Wagon Train	Fish/bound	2009	ND	ND	ND	ND	ND	ND
Pawnee	Fish/bound	2009	0.196	ND	ND	0.18	ND	ND
Rockford	Fish/bound	2009	0.32	0.28	ND	ND	ND	ND
Rockford	Fish/free	2009	0.254	ND	ND	0.55	ND	ND
Rockford	Fish/bound	2010	2.57	1.53	ND	4.13	5.07	ND
Kirkman’s Cove	Fish/free	2010	0.102	0.158	ND	0.23	0.21	ND

**Figure 3:**
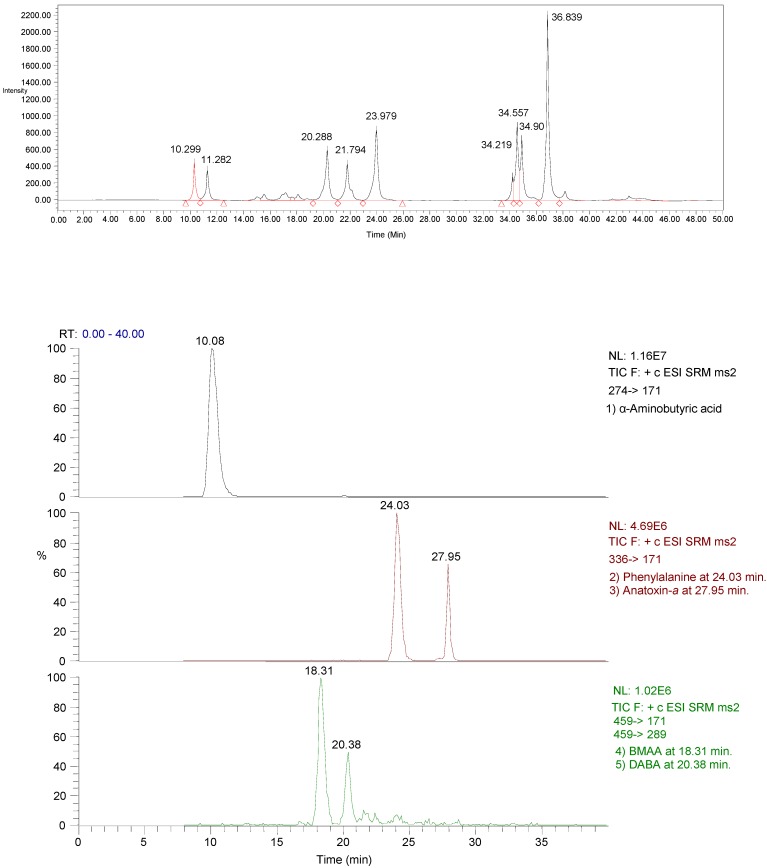
Comparison of HPLC/FD and LC/MS/MS chromatograms for extract from reservoir water samples collected from Rockford reservoir in 2009. LC/MS/MS selected reaction monitoring (SRM) peaks at 10.08, 18.31, 20.38, 24.03, and 27.95 min correspond to detections of α-aminobutyric acid (IS), BMAA, DABA, phenylalanine, and anatoxin-*a*, respectively. Details of HPLC and LC/MS/MS conditions are provided in Al-Sammak *et al*. (2013). Note that extracts were analyzed on different instruments.

### 2.4. LC/MS/MS Confirmation

Because fluorescence detection can result in false positives for complex matrices, cyanotoxin concentrations measured in selected water, fish, and plant samples were confirmed using liquid chromatography tandem mass spectrometry (LC-MS/MS). Extracts from 19 positive and four negative samples obtained using HPLC/FD were subjected to additional analysis using mass spectrometry. LC/MS/MS data are generally comparable to results using fluorescence detection (HPLC/FD) (Table 6). As was expected, the negative samples showed no trace of cyanotoxins, meaning the use of HPLC/FD could be very beneficial as a first-step investigation method, and a good sample screening method [18,25,49,59,65].

BMAA and DABA were detected by using LC-MS/MS in plant seeds of *Cycas revoluta* and *Lathyrus latifolius*, with a concentration of free BMAA of 6.96 µg/g^-1^ and DABA at 4.21 µg/g^−1^ [66]. BMAA and DABA were both detected in Dutch urban waters with cyanobacterial blooms using LC-MS/MS, with free BMAA at 42 µg/g [14]. Jonasson *et al*. [17] detected BMAA in Baltic fish using LC/MS/MS method and Li *et al*. [15] used LC/MS/MS to determine the level of BMAA in Chinese waters. Anatoxin-*a* was also detected by using LC-MS/MS [37,67]. These studies support our use of LC-MS/MS as a necessary confirmation, and the presence of BMAA along with other cyanotoxins such as DABA [25,62] (Figure 3).

## 3. Experimental Section

### 3.1. Sample Collection

**Figure 4:**
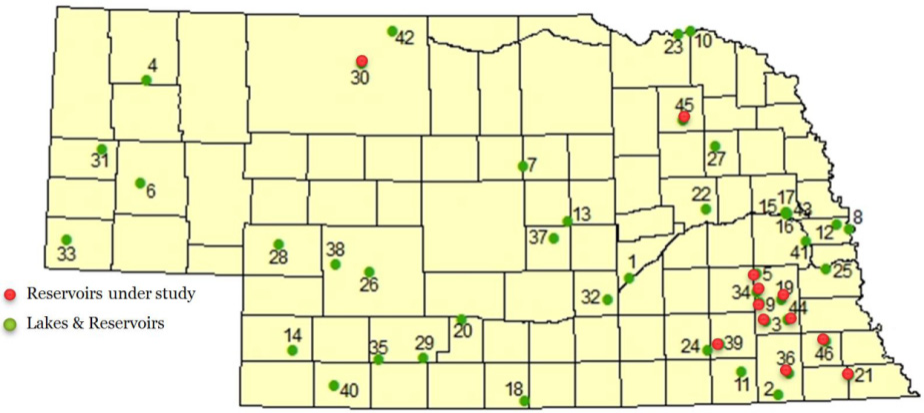
Nebraska map showing reservoirs under study locations for the present study in red dots and other Nebraska Department of Environmental Quality (NDEQ) monitored locations in green.

Between 2009 and 2010, a total of 387 environmental and biological samples were collected from 12 different reservoirs across Nebraska, USA (Figure 4). Ten reservoirs under study were well known for a history of cyanotoxins problems since 2004 [53]. Sample collection was coordinated to occur within 1–3 days after an official lake water quality health alert was announced by Nebraska Department of Environmental Quality (NDEQ), when microcystin levels exceeded 20 µg/L (ppb). According to NDEQ, health alerts remain in effect until microcystin levels decline to below 20 ppb for two consecutive weeks [54]. Two additional reservoirs with no history of cyanotoxins since 2004 in this study were used as controls. All samples were collected in brown polypropylene bottles, transported in a cooler with ice to the Water Sciences Laboratory following USGS guidelines for sampling [68]. Samples then were kept frozen (−20 °C) until extraction and analysis [37].

In 2009 and 2010, a total of 67 (31 in 2009 and 36 in 2010) water samples were collected in 500-mL amber glass bottles with Teflon-lined lids [69] during summer months typical for toxic algal outbreaks in Nebraska lakes. A total of 3–4 samples were collected from each reservoir, taken from different geographic directions, mostly the opposite side to the wind direction. A total of 248 fish samples (115 in 2009, 133 in 2010) were collected from all 12 lakes (Figure 4). Fish samples collected include bottom feeding fish such as carp and catfish using either nets or electrofishing methods with the help of the Nebraska Game and Parks Commission, Fisheries, Southeast District. The 72 aquatic plants samples (36 in 2009, 36 in 2010) collected are well known to grow in Midwestern lakes and commonly known as water milfoil (genus *Myriophyllum*). Three species (*M. aquaticum*, *M. heterophyllum* and *M. spicatum*) have become particularly aggressive invasive plants in lakes, natural waterways and irrigation canals in North America [63,70]. This has prompted the implementation of control plans by many U.S. states most affected by the invasions. These aquatic plants were hand collected and kept frozen in plastic zipper bags until processing.

### 3.2. Sample Extraction and Analysis

Details of method development and validation are published elsewhere [55]. Standards were prepared from pure reagents. DABA (DL-2,4-diaminobutyric dihydrochloride) standards were purchased from Acros organics, NJ, USA, anatoxin-*a* from Tocris Bioscience, Bristol, UK, and L-BMAA (β-*N*-methylamino-l-alanine hydrochloride) and AABA (dl-2-aminobutyric acid) from Sigma-Aldrich, St. Louis, MO, USA. Waters, Oasis-MCX 6cc (150 mg) LP extraction cartridges were purchased from Waters Corp. (Milford, MA, USA). Reagents used in the AQC synthesis included dry acetonitrile (Fisher Scientific, Fair Lawn, NJ, USA), 6-aminoquinoline (AMQ) and Di(*N*-succinimidy) carbonate (DSC) which were obtained from Sigma-Aldrich Co., St. Louis, MO, USA.

Cyanotoxins were extracted from water using Oasis MCX (Waters Corporation, Milford, MA, USA) solid phase extraction cartridges (SPE). The MCX column was conditioned using 5 mL methanol, allowed to dry for at least 10 min, and then washed with 5 mL reagent water. Extracted toxins were eluted using 6 mL of 5% (*v*/*v*) ammonia ammonium hydroxide in methanol, evaporated under nitrogen, dissolved reagent water and derivatized using AQC.

Tissue extracts were prepared using a protocol provided by the Institution of Ethnomedicine in Jackson Hole, WY with some modifications [55]. Frozen fish were thawed and filleted and the aquatic plants were mechanically cleaned. Both fish and plant samples were freeze dried in liquid nitrogen overnight using a Labconco freezer-drying system. Next day samples were ground fine and returned to the freezer. Samples were weighed (0.100 g) into a 15 mL centrifuge tube then mixed with 1 mL of 0.1 N TCA by vortexing for 1 min using a Fisher Scientific Sonic dismembrator. The probe washed between samples with 100% purified water, 50% methanol in water and 100% methanol. The sonicated mixture was stored at 4 °C overnight (16 h) to free protein-bound amino acids. The mixture was then vortexed and centrifuged for 10 min at 1300 rpm to separate solids from the aqueous extract. The extract containing unbound or “free” amino acids, including BMAA and DABA, were transfer extract to microcentrifuge filter tube (Whatman microfilter MWCO) for removal of suspended proteins and centrifuged for 10 min at 1300 rpm. Extraction was repeated by adding 1 mL of 0.1 N TCA to the pellets in the original centrifuge tube, vortexed, sonicated, centrifuged, and filtered before combining with the first portion for subsequent derivatization.

Bound toxins were extracted in the remaining pellet by transferring and mixing with 2 mL of 6 N HCl in a glass centrifuge tube. The toxins in the solid material were released through acid hydrolysis and heated at 110 °C overnight (16 h). After hydrolysis, the suspension was filtered using a microcentrifuge filter (Whatman Ultrafiltration microfilter, EMD Millipore, Billerica, MA, USA) and dried under vacuum for 2 h. After drying, sample filtrate was reconstituted with 1 mL reagent water and derivatized with AQC. Two methods have been used to detect cyanotoxins in our biological samples [55] and are briefly described in the following sections.

#### 3.2.1. HPLC/FL Instrumental Method

High performance liquid chromatography with fluorescence detection (HPLC/FD) was used for preliminary analysis of all extracts. Amino acids and cyanotoxins were separated using a reverse-phase column (Kromasil-Thermohypersil C8 column, 4.6 × 250 mm) on a Waters HPLC Alliance 2695 solvent controller and autosampler (Waters Corporation, Milford, MA, USA). Cyanotoxins concentrations were quantified by detection of the fluorescent tag (Waters 2475 Multi λ-Fluorescence Detector) with excitation at 250 nm and emission at 395 nm with reference to a standard curve. An example chromatogram is show in Figure 3.

#### 3.2.2. LC/MS/MS Instrumental Method

Liquid chromatography-ion trap tandem mass spectrometer (LC/MS/MS) used for confirmation and quantitative analysis of BMAA, DABA, and anatoxin-*a*. Separation used a Waters 2695 solvent controller and auto sampler interfaced with a Finnigan LCQ “Classic” ion trap mass spectrometer equipped with atmospheric pressure ionization fitted with an electrospray ionization (ESI) source (LCQ Classic, Thermo Electron, Waltham, MA, USA). Instrument control, data processing, and analysis used Xcalibur software. A Hypurity C18 HPLC column (2.1 mm × 250 mm × 5 µm; Thermo-Scientific, Waltham, MA, USA) was employed for separation. The mobile phase consisted of solvent A (ammonium formate; 0.5 g/L in water/solvent B (ammonium formate; 0.5 g/L in methanol).

## 4. Conclusions

The cyanotoxins BMAA, DABA, and anatoxin-*a* are reported for multiple Midwestern impoundments. Ninety-four environmental samples with various levels of BMAA, DABA, and anatoxin-a, out of 378 samples, were collected. Water samples showed 31 positives out of 67, fish samples showed 33 positives out of 248, and aquatic plants (water milfoil) displayed 30 positive samples out of 72 collected samples (Table 7). Summer is the main time of the year that cyanobacteria produce/release cyanotoxins into the environment. The occurrence of cyanotoxins in lake water, aquatic plants and fish suggests potential for transfer throughout the ecosystem to higher levels in the food web. As cyanotoxins may reach higher organisms such as humans through these additional pathways, there is the potential for damage leading to neurodegenerative diseases such as ALS, PD, and AD. Thus, it is important to recognize these potential routes of exposure; directly by drinking or swimming in water or indirectly by eating contaminated fish.

**Table 7:** Summary of samples with positives and undetected (ND) levels of cyanotoxins from a total of 387 samples collected between 2009 and 2010.

Samples	2009	2010	Total positive (% of total)	ND
Reservoir water	13	18	31 (46.3)	36
Fish	24	9	33 (13.3)	215
Aquatic plant	15	15	30(41.7)	42
Total samples collected in 2009–2010 = 387			94	293

Of the 12 Nebraska reservoirs sampled in this study, two reservoirs did not have detectable levels of cyanotoxins and served as a control group in the study. This is consistent with previous monitoring for cyanotoxins in Nebraska. BMAA, DABA, and anatoxin-*a* were detected in Nebraska reservoirs in 2009–2010 samples, including fish, aquatic plant, and lake water. BMAA and DABA were measured in 13 reservoir water samples in 2009 and 12 samples in 2010, while anatoxin-*a* was measured in 13 samples in 2009 and 18 samples in 2010. BMAA was found in 22 fish tissue samples in 2009 and nine samples in 2010, while DABA was detected in 19 samples in 2009 and seven samples in 2010. Anatoxin-*a* was not detected in any fish samples. BMAA, DABA, and anatoxin-*a* were detected in 15 samples of aquatic plants collected in 2009 and 15 in 2010 (Table 7 and Table 8).

**Table 8:** Cyanotoxins detection summary for Nebraska reservoir samples from 2009 and 2010.

Cyanotoxins	Water		Fish		Plant		Percent of detections
	2009	2010	2009	2010	2009	2010	2009–2010
BMAA	13	12	22	9	5	5	18%
DABA	13	12	19	7	5	5	17%
Anatoxin-*a*	13	18	0	0	5	5	12%
Total samples	31	36	115	133	36	36	387

The results suggest that detectable reservoir water levels of BMAA, DABA, and anatoxin-*a* may be associated with a detectable levels of cyanotoxins in fish tissue and aquatic plants. We can also hypothesize that the presence of such cyanotoxins in Nebraska reservoir water will transfer in the food web with an increased potential for bioaccumulation. Because of variable occurrence of DABA and anatoxin-*a*, these cyanotoxins may not be produced by the same cyanobacteria species. BMAA concentrations were more clearly associated with detection of microcystins.
